# The Minhang Pediatric Biobank cohort study: protocol overview and baseline characteristics

**DOI:** 10.1186/s12887-024-04763-6

**Published:** 2024-04-27

**Authors:** Xiaosa Wen, Xinyue Zhang, Yun Qiu, Yaqin Wang, Liujie Zhu, Tao Liu, Zengliang Ruan

**Affiliations:** 1 Minhang District Center for Disease Control and Prevention, Shanghai, China; 2 Key Laboratory of Environmental Medicine and Engineering of Ministry of Education, and Department of Epidemiology & Health Statistics, School of Public Health, Southeast University, 87 Dingjiaqiao, Gulou District, Jiangsu, Nanjing, 210096 China; 3 Department of Public Health, School of Medicine, Nanjing University of Chinese Medicine, Nanjing, China; 4 School of Health and Life Sciences, University of Health and Rehabilitation Sciences, Qingdao, China; 5 Department of Biochemistry and Molecular Biology, School of Medicine, Southeast University, Jiangsu, Nanjing, 210096 China; 6 Jiangsu Provincial Key Laboratory of Critical Care Medicine and Department of Critical Care Medicine, School of Medicine, Zhongda Hospital, Southeast University, Nanjing, China; 7 Department of Epidemiology, School of Public Health, Sun Yat-Sen University, Guangzhou, China; 8 Department of Medical Epidemiology and Biostatistics, Karolinska Institutet, Stockholm, Sweden

**Keywords:** Pediatric biobank, Child cohort, Electronic medical record, Healthcare, Physical examination

## Abstract

**Background:**

Little has been done to establish biobanks for studying the environment and lifestyle risk factors for diseases among the school-age children. The Minhang Pediatric Biobank (MPB) cohort study aims to identify factors associated with health and diseases of school-aged children living in the urban or suburban area of Shanghai.

**Methods:**

This population-based cohort study was started in all sub-districts/towns of Minhang district of Shanghai in 2014. First-grade students in elementary school were enrolled during the time of their routine physical examinations, with self-administered questionnaires completed by their primary caregivers. Additional information was extracted from multiple health information systems. Urine and saliva samples were collected during the baseline survey and follow-up visits.

**Results:**

At the end of 2014 academic year, a total number of 8412 children and their parents were recruited, including 4339 boys and 4073 girls. All the participants completed the baseline survey and physical examination, and 7128 urine and 2767 saliva samples were collected. The five most prevalent childhood diseases in this population were dental caries, bronchitis, pneumonia, asthma and overweight/obese.

**Conclusions:**

The MPB cohort has been successfully established, serving as a useful platform for future research relating to the genetic, environmental and lifestyle risk factors for childhood diseases.

**Supplementary Information:**

The online version contains supplementary material available at 10.1186/s12887-024-04763-6.

## Background

During the past decades, the rapid industrialization and urbanization have led to increased exposure to environmental pollution, changes in dietary structure and lifestyles, and health disparities [1]. These factors have further caused a number of public health challenges, especially the remarkable increment in the prevalence rates of common childhood diseases such as obesity, diabetes, myopia and dental caries [2–4]. Thus, the relationships between environmental or lifestyle risk factors and the health and diseases of children have attracted a great attention.

As a great step to advance the informatization of medical resources in Shanghai, the district government launched the Health Information Platform (HIP) in 2010, based on a management system of resident’s Electronic Health Record (EHR), Electronic Medical Record (EMR) and public service information network. The EHR includes the resident’s demographic information, health status and disease, medical history, while the EMR mainly incorporated a Healthcare Data System (HDS) and a Community Health Service Information System (CHSIS). The HDS implemented both a data exchange center and a comprehensive resident’s health record system, while the CHSIS encompassed a more extensive array of modules, such as the general practice diagnosis and treatment module, child healthcare services, planned immunization procedures and a physical examination system. Furthermore, the HIP was intentionally structured to connect with a biobank tasked with the collection and curation of diverse human biological specimens within the local area.

A biobank is conventionally characterized as a specialized repository designed to store and administrate human biological samples (such as tissue, blood, saliva, urine, cells, protein, DNA and RNA), commonly referred to as biospecimens, along with relevant information organized in a systematic way for medical and biological sciences research [5, 6]. These resources are curated with the primary objective of facilitating and supporting research endeavors in the realms of medical and biological sciences. The rapid advancement of molecular technology and the evolution of translational medicine have significantly catalyzed the proliferation of biobanks, with the UK Biobank, Danish National Biobank and China Kadoorie Biobank standing out prominently [7–9]. Moreover, a multitude of biobanks, each focusing on distinct pediatric ailments, have been documented on both national and global scales [10, 11]. For example, numerous biobanks dedicated to acquiring tissue samples from pediatric populations have been documented within Western nations, such as the Tumor Bank of the Children's Hospital at Westmead, Australia [12] and the Childhood Cancer and Leukemia Group Tumor Bank in UK [13]. Given the escalating necessity for a robust platform for the investigation of health and disease in school-aged children in China, the establishment of pediatric biobank could provide access to a comprehensive repository of biological specimens and associated individual data. Data from laboratory analyses of biological samples can also be linked to various environment, behavior and lifestyle factors extracted from different databases such as EHR, as well as health outcomes from the EMR system, which might help researchers discover potential factors associated with children’s health and diseases.

However, most of the established biobanks in China only collected adult sample [9, 14], and there were limited biobanks focused on school-age children. Therefore, the Minhang Pediatric Biobank (MPB) Project, a prospective cohort study, was set up to investigate the role of environmental and lifestyle factors associated with diseases or other health outcomes (such as metabolomic and microbial profiles) of school-aged children living in an urban or suburban area of Shanghai.

## Methods

### Study site and design

**Fig. 1:**
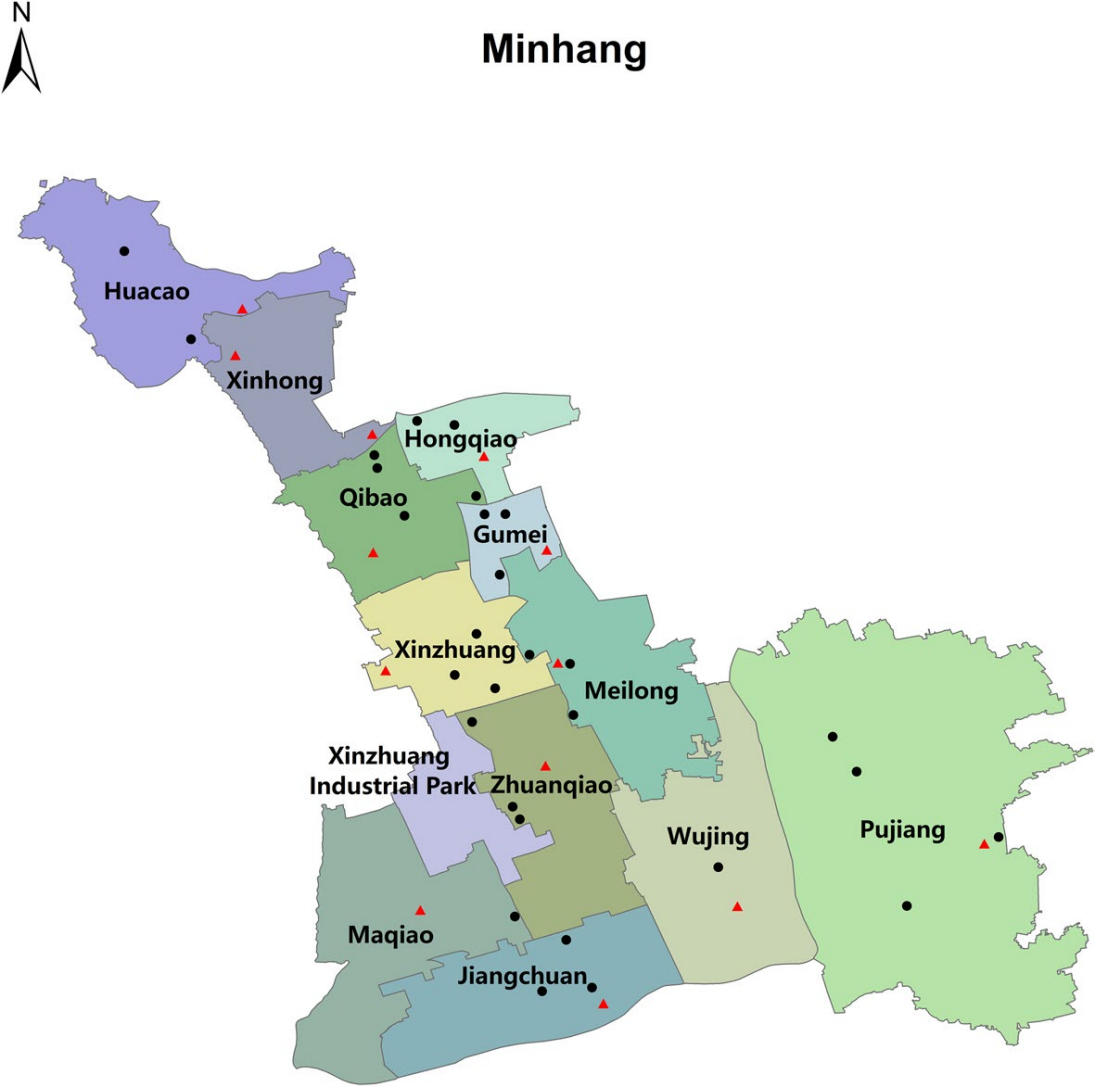
Geographical location of the study area. Black dots indicate the distribution of schools earmarked for questionnaire investigation and urine collection, while red triangles represent the selected schools for additional saliva collection. The original Longbai sub-district has been revoked and the corresponding geographic area now falls under the jurisdiction of Xinhong, Hongqiao and Qibao

Minhang district, shaped like a key, is situated in the hinterland of the Shanghai Municipality with a land area of 372.56 square kilometers (Fig. 1, at approximately 31.071° N latitude and 121.405° E longitude). It has a population of 2,689,000 residents in 2022, with the life expectancy of residents reached 83.67 years and per capita disposable income of 82,400 yuan. Minhang district administers four sub-districts, nine towns and a municipal industrial zone, and the district government is in Xinzhuang Town. While primarily characterized as a residential district, it also hosts many factories and production facilities. It has a typical north subtropical marine monsoon climate with four distinct seasons. The average annual temperature is around 17.1 °C, with the lowest on average in January at around 4.9 °C and the highest in July at around 28.8 °C. The average annual rainfall is around 1,180.7 mm, and July is the wettest month. It also suffers from extreme meteorological conditions including gale, thunderstorms, typhoons, hail, heat and cold waves [15].

**Fig. 2:**
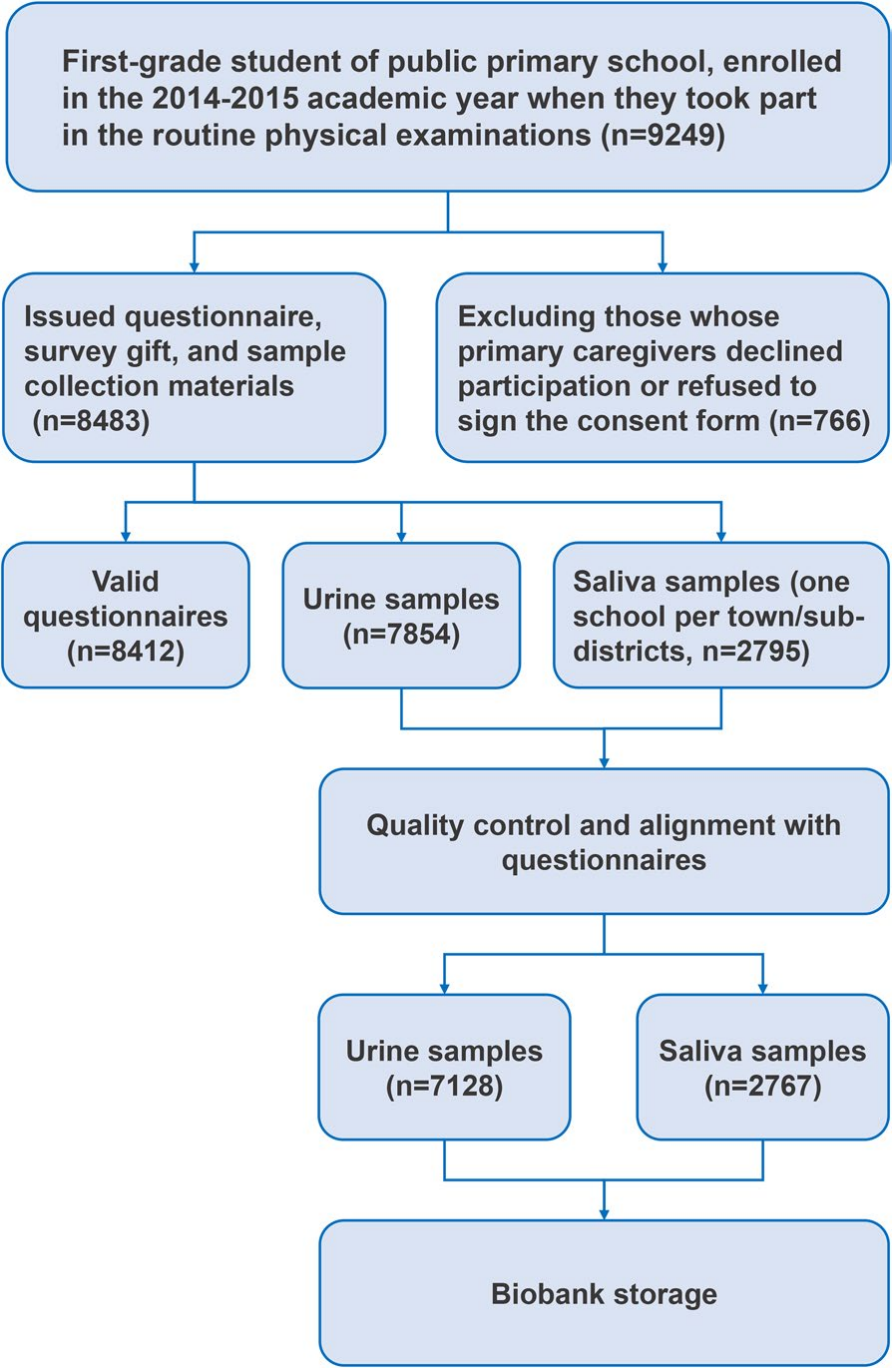
Flowchart of the Minhang Pediatric Biobank Project

With the cooperation of the Health Commission and Education Commission of Shanghai Municipal Government, the MPB project was a multi-community, prospective cohort study which was carried out to investigate the impacts of family and school environment on children’s health. The MPB cohort study was started in September 2014 in all sub-districts/towns of Minhang district, and non-disabled first grade students in 42 public elementary schools were enrolled during the time of school-aged children’s routine physical examinations (Fig. 2).

### Data collection

During the baseline survey, children’s parents or other primary caregivers were asked to complete a self-administered questionnaire regarding both the parents’ and children’s information (Table 1). In the parents’ section, we asked about their socio-demographic characteristics (such as sex, age, occupation, and education level), anthropometry data (including heights and weights), living conditions, exposures, genetic disorders and medical history. In the school-aged children’s section, we collected data on their demographic characteristics (such as sex, birth date), living environment, lifestyle factors (such as dietary behaviors, physical activities, sleep patterns) and medical history. The children’s average exercise duration and intensity were collected and classified as low, moderate, or high based on the perception of their primary caregivers. The physical examination items mainly included: general body check-up; ear, nose, throat, eye and oral examination; general measurements (height, weight, waist circumference, hip circumference and blood pressure); routine blood and urine test; intestinal parasite infections. Data on the diagnosis and treatment of childhood diseases were extracted from the student’s EMR, and additional information was obtained from the students' EHR, which also included a physical examination system that recorded the results of their routine physical examinations. Data from these systems could be matched and double-checked through a unique identification number. These digital work platforms and systems were set up and maintained by Hangzhou Chuangye Software Co. Ltd and Shanghai Avantech BioScience Co., Ltd.

**Table 1:** Data collected during the survey^a^

Items	Measurements
Demographics (Parents and/or children)	Age, sex, ethnic group, occupation, education level, health status, birth weight, school, grade, class, number of siblings
Family	Family structure, history of family diseases
Medical record	Dental caries, anemia, overweight/obese, myopia, farsightedness, amblyopia, bronchitis, pneumonia, asthma, accidental injury
Physical examination	General body check-up; ear, nose, throat, eye and oral examination
Anthropometry data	Height, weight, waist circumference, hip circumference and blood pressure
Living conditions	House type, passive smoking exposure, house renovation, floor board, furniture, pet ownership, air conditioning, ventilation, cooking and heating fuel
Dietary behavior and lifestyles	Dietary habits, exercise, outdoor activities, homework, watching TV, sleep time and quality, tooth brushing
Bioanalysis	Routine blood and urine test; intestinal parasite infections

^a^Data were obtained from multisource included the questionnaire, personal health record and electronic medical record

### Exposures and outcomes

In our future studies, a series of measurements can be used as exposure variables, which might include aforementioned variables that we collected through self-administered questionnaires, physical examinations, and clinical and laboratory indicators recorded in multiple health information systems. In addition, based on ground-based monitors’ measurement and remote sensing estimation, we will also collect environmental data such as air pollution, meteorological factors and residential greenness. Furthermore, the major outcome variables might be the health status collected either by self-reported or extracted from the aforementioned multiple health information systems, observed (such as movement fluctuations seen by a caregiver or healthcare provider), or measured clinically (physical examination, imaging, laboratory testing). For example, children newly diagnosed with asthma, pneumonia, obesity, or myopia during the follow-up periods. In addition, since we have collected the children’s urine and saliva samples, urine metabolites, saliva metabolites and saliva microbiome may also serve as important outcome indicators.

### Follow-up investigation

The follow-up investigation of the MPB study was conducted by community healthcare experts and encompasses a series of components, such as annual physical examinations of the children, the biennial questionnaire survey designed to collect data regarding exposures to diverse risk factors and the measurement of outcomes, alongside the concurrent samples collection during the biennial survey. Moreover, the students' survey data were linked to their EHR and EMR databases and biological samples by a unique number in each students’ personal healthcare card. The community healthcare professionals were asked to work at school twice a week to promote their routine healthcare management, sample collection and follow-up investigation of participants.

### Sample collection

The urine samples from all participants were collected during the baseline and biennial in-person surveys. Before urine collection, the community healthcare experts explained the MPB sample collection requirements to the children and their parents. Each participant was provided with sample collection materials (including one urine collecting container, two standard urine collecting tubes, two ice packs and one sealing bag) intended for the collection of morning urine samples. Two tubes of 10-ml fasting urine samples from each participant were collected in the morning with the help of their parents, then the tubes were immediately put into a sealing bag with two ice packs to maintain a cold environment. The urine samples were delivered to school within 2 h after collection, and the community healthcare professionals were responsible to check the integrity of labels, quality of urine samples and transporting them to the laboratory of Minhang district center for disease control and prevention (MCDC). The total process of transportation and sub-packing was required to be completed within 4 h.

Furthermore, a total of 13 public elementary schools were selected through stratified cluster sampling, with one school chosen per sub-districts/town, saliva samples were then collected exclusively from participants within these selected schools. There are two selecting schools in Xinhong sub-district because the original Longbai sub-district has been revoked, and the corresponding sampling school in Longbai is now under the jurisdiction of Xinhong after the adjustment. During the morning break between classes (about 9:30 a.m. to 10:30 a.m.), each participant was asked to rinse their mouth with water, then 5 mL of saliva samples were collected by spitting into the tube under non-irritating conditions. The collection process was supervised and guided by a specially assigned staff.

The transportation of biospecimen was conducted by a cold-chain logistic company under the supervision of staff from MCDC. Then each of the samples was sub-packed into 1.5-ml sterile pp tubes (enzyme free) with ice packs or crushed ice on a sterile super clean bench and then stored in -80 °C refrigerators. The children’s questionnaires, physical examination results, EMR and biological samples were matched by a unique scanned bar code.

### Quality control

The investigators, including the school doctors, community healthcare professionals and staff of MCDC, were trained prior to the implementation of our study. Standardized work flow was applied under the supervision of staff from MCDC during the whole processes of survey, physical examination, sample collection, transportation, sub-packing and storage to ensure the consistency and accuracy of the study. The samples were transported by a professional cold-chain logistic company, and the total process of sample collection, transportation and sub-packing were required to be completed within 4 h. In order to prevent unnecessary damage to the biological samples caused by circuit issues, the MPB adopted both a dual circuit control and power-off alarming system. Moreover, the storage samples were randomly selected for quality check after one week and every six months thereafter. Additionally, scanned bar codes were adopted to match the children’s questionnaire, EHR, EMR and biospecimen, and data were updated simultaneously to the systems to reduce latency.

### Statistical analysis

The characteristics of participants were described as N (%) for categorical variables, and mean (SD) for continuous variables. In our future analysis plan based on this cohort, we will use mixed-effects models to examine the associations between different outcome measurements and different exposures. For example, linear mixed models will be used to examine the associations between exposures and continuous outcome variables, mixed effects logistic regression models will be used to check the associations between exposures and binary outcome variables, while mixed effects cox regression models will be used to model survival data. Results of the association analyses will be reported as effect estimates and confidence intervals. Given that the censoring of participants does not inherently entail the dismissal of their information, but rather their data might encompass significant insights pivotal to establishing associations, we intend to employ the subsequent four methodologies to address censored data: complete-data analysis; imputation methods such as multiple imputation and random-forest-based imputation; the likelihood-based approach or dichotomizing the data. Other innovative methods such as censored network estimation, maximum likelihood estimation and inverse probability of censoring weighting may also be used [16]. The method should be selected cautiously according to the study aims and missing patterns.

The covariates in the multivariate regression models will be selected mainly using directed acyclic graphs [17]. We may also identify the potential confounders based on their associations with the outcomes of interest or a change in effect estimate of more than 10% [18, 19]. In addition, some demographic variables such as sex, age and household income may also be included as controlling variables. The values of potential confounders may change over time leading to time varying confounding, which will be adjusted for by conventional methods such as time dependent Cox regression, generalized estimating equations, or random effects models, and causal methods such as inverse-probability-of-treatment weighting, G estimation, or the parametric G formula [20].

In future analyses, a variety of sensitivity analyses will be designed to confirm the stability of the associations between different exposure and outcomes under various specifications of eligibility criteria. For example, the selection of various lengths of data availability over the study period, the additional inclusion or exclusion of different covariates, or examining the associations between exposures and outcomes of interest with multiple analysis models. Further sensitivity association analyses will be restricted to individuals with complete data on the key exposure and outcome variables. The statistical analyses will be conducted mainly by advanced version of R package, and a two-tailed *p*-value below 0.05 will be considered statistically significant.

## Results

**Fig. 3:**
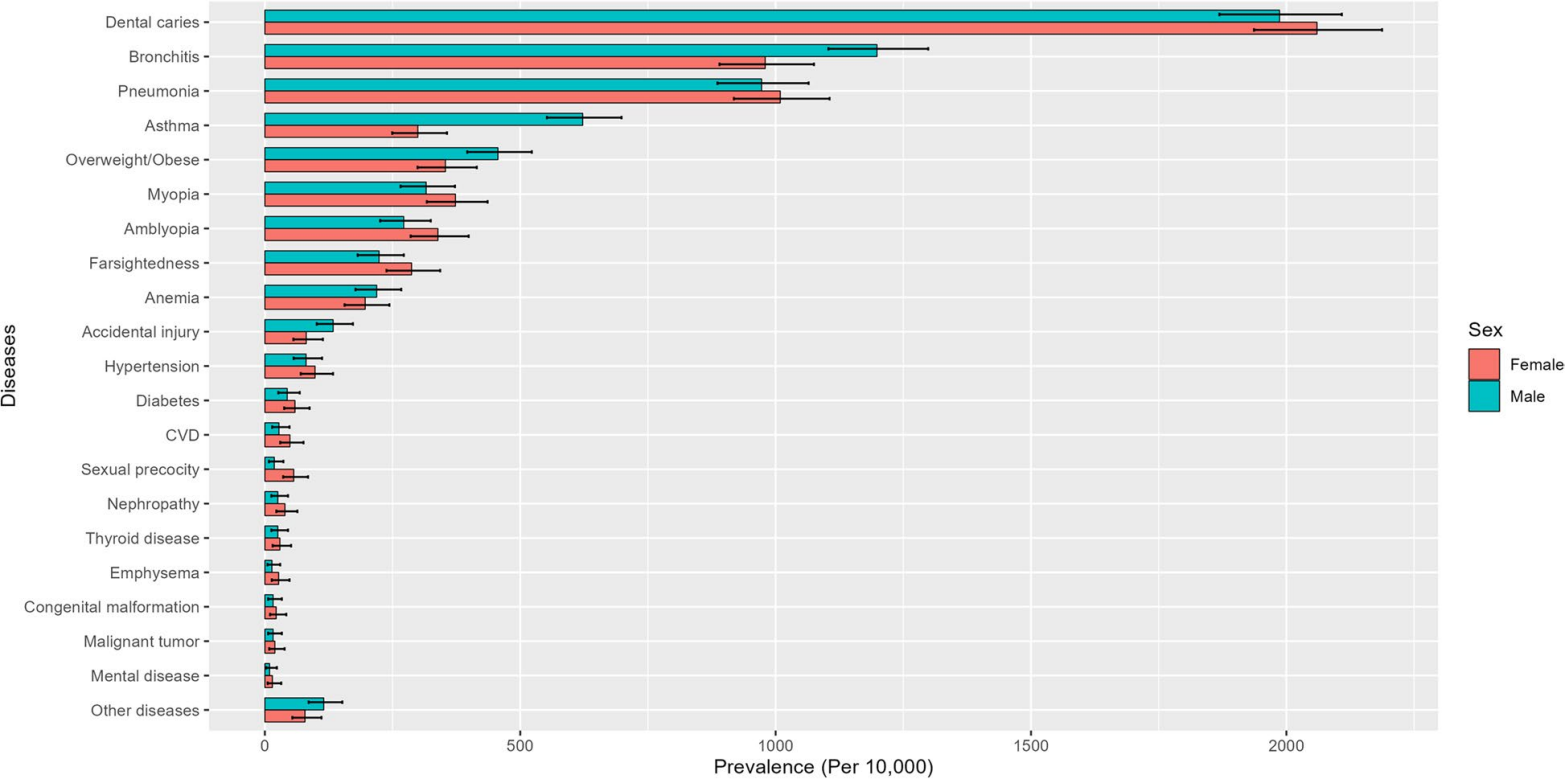
Prevalence of diseases among the participants of Minhang Pediatric Biobank

At the end of 2014 academic year, we recruited a total number of 8412 children from 42 public primary schools, including 4339 boys and 4073 girls. The baseline characteristics of the children were shown in Table 2. The mean age of all school-aged children was 6.64 ± 0.29 years, with 6.65 ± 0.28 and 6.64 ± 0.29 years for boys and girls, respectively. These children had an average of siblings of 1.39. The mean height, weight, BMI were 123.89 ± 5.39 cm, 25.15 ± 5.17 kg, 16.27 ± 2.54 kg/m^2^, and the mean waist and hip circumference were 55.07 ± 6.68 and 65.39 ± 6.07 cm. A total of 4011 (47.68%) of the participants were exposed to passive smoking at home, 7308 (86.88%) had moderate exercise; 7778 (92.46%) children used screen devices less than 2 h per day; 3378 (40.16%) children had a very good sleep quality and the most common sleep time was between 9 to 10 h [5695 (67.70%)]; 1755 (20.86%) children had house renovation during the past year; 1502 (17.86%) children had pets in their family; 7556 (89.82%) families had ventilation every day; natural gas was the most important fuel for cooking and heating [6738 (80.10%)]. In addition, baseline characteristics of the participants divide by the seven diseases with the highest prevalence were presents in Table S1. During the baseline investigation, we collected 7128 urine and 2767 saliva samples (Table 3). In addition, the prevalence of common diseases among MPB participants were shown in Fig. 3. For example, the prevalence of dental caries, bronchitis, pneumonia, asthma, overweight/obese, myopia, amblyopia, farsightedness and anemia of the participants were 20.22% (95% CI: 19.37%, 21.10%), 10.92% (95% CI: 10.27%, 11.61%), 9.90% (95% CI: 9.27%, 10.56%), 4.66% (95% CI: 4.22%, 5.13%), 4.07% (95% CI: 3.65%, 4.51%), 3.44% (95% CI: 3.06%, 3.85%), 3.04% (95% CI: 2.69%, 3.43%), 2.54% (95% CI: 2.22%, 2.90%) and 2.08 (95% CI: 1.79%, 2.41%), respectively.

**Table 2:** Baseline characteristics of the participants^a^^,^^b^

Characteristics	Sex		Total
	**Male**	**Female**	
**Age, years**	6.65 (0.28)	6.64 (0.29)	6.64 (0.29)
**Number of siblings**	1.38 (1.07)	1.40 (1.06)	1.39 (1.07)
**Height**	124.64 (5.29)	123.07 (5.38)	123.89 (5.39)
**Weight**	26.08 (5.42)	24.14 (4.69)	25.15 (5.17)
**BMI**	16.68 (2.65)	15.83 (2.33)	16.27 (2.54)
**Waist circumference**	56.46 (7.03)	53.58 (5.93)	55.07 (6.68)
**Hip circumference**	66.23 (6.45)	64.49 (5.49)	65.39 (6.07)
**Passive smoking exposure**			
Yes	2082 (24.75)	1929 (22.93)	4011 (47.68)
No	2230 (26.51)	2113 (25.12)	4343 (51.63)
**Exercise**			
Low	367 (4.36)	391 (4.65)	758 (9.01)
Moderate	3778 (44.91)	3530 (41.96)	7308 (86.88)
High	169 (2.01)	122 (1.45)	291 (3.46)
**Screen use**			
≤ 2 h	3981 (47.33)	3797 (45.14)	7778 (92.46)
> 2& ≤ 4 h	273 (3.25)	194 (2.31)	467 (5.55)
> 4& ≤ 6 h	12 (0.14)	13 (0.15)	25 (0.30)
> 6 h	4 (0.05)	7 (0.08)	11 (0.13)
**Sleep quality**			
Very good	1759 (20.91)	1619 (19.25)	3378 (40.16)
Good	1899 (22.57)	1855 (22.05)	3754 (44.63)
Average	645 (7.67)	567 (6.74)	1212 (14.41)
Bad	18 (0.21)	17 (0.20)	35 (0.42)
**Sleep time**			
≤ 9 h	1076 (12.79)	1046 (12.43)	2122 (25.23)
> 9& ≤ 10 h	2953 (35.10)	2742 (32.60)	5695 (67.70)
> 10& ≤ 11 h	268 (3.19)	258 (3.07)	526 (6.25)
> 11 h	12 (0.14)	5 (0.06)	17 (0.20)
**House renovation during the past year**			
Yes	898 (10.68)	857 (10.19)	1755 (20.86)
No	3416 (40.61)	3185 (37.86)	6601 (78.47)
**Pet ownership**			
Yes	729 (8.67)	773 (9.19)	1502 (17.86)
No	3579 (42.55)	3272 (38.90)	6851 (81.44)
**Ventilation**			
Everyday	3914 (46.53)	3642 (43.30)	7556 (89.82)
1 to 3 times/week	346 (4.11)	345 (4.10)	691 (8.21)
1 to 5 times/month	39 (0.46)	48 (0.57)	87 (1.03)
Seldom	17 (0.20)	17 (0.20)	34 (0.40)
Never	2 (0.02)	1 (0.01)	3 (0.04)
**Cooking and heating fuel**			
Coal gas	353 (4.20)	339 (4.03)	692 (8.23)
Natural gas	3485 (41.43)	3253 (38.67)	6738 (80.10)
Liquefied petroleum gas	233 (2.77)	225 (2.67)	458 (5.44)
Electricity	146 (1.74)	129 (1.53)	275 (3.27)
Coal	6 (0.07)	6 (0.07)	12 (0.14)
Others	4 (0.05)	4 (0.05)	8 (0.10)

^a^Data are presented as mean (SD) or number (%)

^b^Missing (n): Passive smoking exposure (58); Exercise (55); Screen use (131); Sleep quality (33); Sleep time (52); House renovation during the past year (56): Pet ownership (59); Ventilation (41); Cooking and heating fuel (229)

**Table 3:** The number of schools, participants, urine and saliva samples from different sub-districts/towns and selected schools

Towns/ sub-districts	Number of schools	Number of participants	Urine samples (10 mL/Person)	Selected schools to collect saliva	Saliva samples (5 mL/Person)
**Gumei**	4	685	542	Gumei School	96
**Hongqiao**	1	138	132	Hongqiao Primary School	138
**Huacao**	3	529	422	Huacao Primary School	178
**Jiangchuan**	4	399	200	Huaping Primary School	32
**Longbai**^a^	5	891	761	Hanghua No.1 Primary School	174
**Maqiao**	2	284	184	Qiangshu Primary School	147
**Meilong**	4	1043	957	Minhang Experimental Primary School (Chun Cheng)	365
**Pujiang**	5	962	898	Pujiang No. 2 Primary School	235
**Qibao**	3	1037	965	Mingqiang Primary School (West Campus)	227
**Xinzhuang**	4	929	738	Kangcheng School	272
**Wujing**	2	330	213	Zizhu Primary School	185
**Xinhong**	1	115	114	Minhang Experimental School Affiliated to Shanghai International Studies University	115
**Zhuanqiao**	4	1070	1002	Tianyuan Foreign Language Primary School (Jin Du)	603
**Total**	42	8412	7128	13	2767

^a^The Longbai sub-district has been revoked and the corresponding geographic area now falls under the jurisdiction of Xinhong, Hongqiao and Qibao

## Discussion

The MPB cohort was a multi-community prospective study aiming to investigate the effects of demographic, environmental and lifestyle risk factors for childhood diseases in different circumstances. We could also have comprehensive evaluations of the metabolomic and microbial factors related to child health by collecting urine and saliva samples from the school-aged children. The establishment of electronic linkage with EHR and EMR databases in our study increased the range and reliability of our data.

The MPB was established under the cooperation of healthcare and educational departments of the government, which might be helpful for the follow-up management. The collection of samples by introducing community healthcare experts to school twice per week and working with the school doctor ensured the whole-course health management of the children. Furthermore, the interlinkage of diverse health-related systems within our study has yielded a range of benefits in monitoring the health and disease statuses of the children. This integration amalgamates accessible health information encompassing diagnoses, treatments, and prognostic factors. This not only culminates in cost reduction through the concurrent updating of critical data, but also significantly enhances both the follow-up rate and the precision of outcome measurements.

School children are in a critical phase of their lifespan characterized by paramount physical growth and development [21, 22]. In this stage, children are very vulnerable to environmental risk factors, making early diagnosis and intervention of diseases more advantageous than the adult population [23]. The establishment of MPB provides an important platform to assess different factors associated with health and disease of children, which will be crucial for pediatric biomedical research in the future. The linkage of the biobank, EHR and EMR of the children has also facilitated the large-scale data analysis from different sources, which has significant advantages compared to similar studies conducted in the past [24]. In addition, the biobank also enables us to explore the interaction effects of environmental and lifestyle factors on specific diseases, thereby enhancing the health management and promotion of the school-aged children.

Although it was not feasible to conduct particular descriptive analyses for all the information collected in this manuscript, our results suggested that a high percentage of children were exposed to passive smoking at home, which was corresponding to a previous result that about 59.4% male participants in Songjiang and Jiading district of Shanghai were smokers [25]. In addition, our results showed that the participants generally had moderate screen use and good sleep quality, which were similar to that of previous comparable studies [26]. Moreover, our findings indicated that dental caries, bronchitis, pneumonia, asthma and overweight/obese were the top five prevalent primary childhood diseases, consistent with previous studies [27–29]. This suggests that we should pay more attention on discovering more public health measures to reduce the prevalence of these diseases and burdens caused in the future.

Measures have been taken to protect the samples from degradation. For example, the samples underwent minimal processing and were transported by a cold-chain logistic deliver company at 4 °C before being delivered to the laboratory of MCDC within four hours of collection. The samples were sub-packed in the laboratory and stored at − 80 °C. According to previous reports, biological sample could stay stable for a long time at − 80 °C, including those readily degradable molecules such as cytokines [7]. In addition, despite previous studies have provided a guarantee of reliability and stability [30], the storage samples were randomly selected for quality check due to the importance of sample integrity.

In order to achieve a cost-effectively recruitment and to target regions with both urban and suburban area, our study was not designed to enroll a national representative population of China. However, the inclusion of relatively large number of children from different sub-districts/towns could help us discover important new findings on the cause of common pediatric diseases which might be generalizable to children of the whole country. In addition, this study only enrolled non-disabled students from public schools, which might introduce a selection bias. Furthermore, the MPB collected urine and saliva samples of school-aged children rather than serum samples. This was mainly due to that the venous blood collection was an invasive method, which was difficult to conduct in children because of the physiological characteristics in veins of their hands and parental concerns. However, according to previous reports, the urine samples were easier to collect, transport and long-term storage. Additionally, both urine and saliva samples contain a large number of enzymes and hormones relating to the metabolism, growth and development of children [31, 32].

## Conclusions

In conclusion, the inception of MPB cohort study has furnished an invaluable foundation for the advancement of subsequent investigations pertaining to genetic, environmental and lifestyle risk factors implicated in childhood ailments. This initiative is poised to yield substantial economic and societal dividends within the substantial realms of pertinent research domains.

### Supplementary Information

**Supplementary Material 1. **

## Data Availability

The datasets used and/or analyzed during the current study are available from the corresponding author on reasonable request.

## Abbreviations

MPB: Minhang pediatric biobank
HIP: Health information platform
EHR: Electronic health record
EMR: Electronic medical record
HDS: Healthcare data system
CHSIS: Community health service information system
MCDC: Minhang district center for disease control and prevention
