# Supplementary material for: The Minhang Pediatric Biobank cohort study: protocol overview and baseline characteristics
Source: BMC Pediatr. 2024 Apr 27;24:282. doi: 10.1186/s12887-024-04763-6 (PMC11055290; doi:10.1186/s12887-024-04763-6)
Supplement: Supplementary file 1 — Supplementary Material 1. [file 12887_2024_4763_MOESM1_ESM.pdf]

Table S1. Baseline characteristics of the participants divide by the seven diseases with the highest prevalence [†,‡].

Characteristics	Disease							Total
	Dental caries	Bronchitis	Pneumonia	Asthma	Obese/ Overweight	Myopia	Amblyopia	
**Age, years**	6.65 (0.26)	6.64 (0.26)	6.63 (0.26)	6.65 (0.28)	6.68 (0.26)	6.61 (0.43)	6.63 (0.26)	6.64 (0.29)
**Number of siblings**	1.31 (0.67)	1.30 (0.57)	1.29 (0.93)	1.30 (0.56)	1.28 (0.56)	1.30 (0.59)	1.36 (0.70)	1.39 (1.07)
**Height**	123.81 (5.39)	123.89 (5.67)	123.93 (5.53)	124.9 (5.15)	127.35 (5.55)	123.73 (5.59)	123.43 (5.42)	123.89 (5.39)
**Weight**	24.85 (5.14)	25.15 (5.15)	25.10 (5.13)	26.20 (5.39)	32.72 (6.06)	24.85 (5.08)	25.04 (5.12)	25.15 (5.17)
**BMI**	16.11 (2.48)	16.27 (2.45)	16.25 (2.46)	16.66 (2.57)	20.07 (2.81)	16.10 (2.50)	16.30 (2.51)	16.27 (2.54)
**Waist circumference**	54.46 (6.49)	55.01 (6.87)	54.97 (6.78)	56.48 (6.96)	63.98 (7.90)	54.54 (6.71)	54.66 (6.89)	55.07 (6.68)
**Hip circumference**	65.06 (5.90)	65.25 (6.27)	65.31 (5.81)	66.69 (5.93)	73.00 (7.72)	64.59 (7.00)	65.41 (6.05)	65.39 (6.07)
**Sex**								
Male	862 (50.68)	520 (56.58)	422 (50.66)	270 (68.88)	198 (57.89)	137 (47.40)	118 (46.09)	4339 (51.58)
Female	839 (49.32)	399 (43.42)	411 (49.34)	122 (31.12)	144 (42.11)	152 (52.60)	138 (53.91)	4073 (48.42)
**Passive smoking exposure**								
Yes	777 (45.68)	461 (50.16)	399 (47.90)	193 (49.23)	192 (56.14)	141 (48.79)	130 (50.78)	4011 (47.68)
No	921 (54.14)	456 (49.62)	427 (51.26)	198 (50.51)	150 (43.86)	148 (51.21)	126 (49.22)	4343 (51.63)
**Exercise**								
Low	151 (8.88)	109 (11.86)	106 (12.73)	49 (12.5)	37 (10.82)	28 (9.69)	38 (14.84)	758 (9.01)
Moderate	1497 (88.01)	774 (84.22)	692 (83.07)	327 (83.42)	287 (83.92)	247 (85.47)	211 (82.42)	7308 (86.88)
High	49 (2.88)	33 (3.59)	34 (4.08)	15 (3.83)	17 (4.97)	9 (3.11)	7 (2.73)	291 (3.46)

**Screen use**								
≤2h	1614 (94.89)	863 (93.91)	787 (94.48)	362 (92.35)	324 (94.74)	271 (93.77)	242 (94.53)	7778 (92.46)
>2&≤4h	75 (4.41)	41 (4.46)	37 (4.44)	22 (5.61)	15 (4.39)	15 (5.19)	11 (4.30)	467 (5.55)
>4&≤6h	3 (0.18)	4 (0.44)	4 (0.48)	3 (0.77)	2 (0.58)	1 (0.35)	0 (0.00)	25 (0.30)
>6h	0 (0.00)	2 (0.22)	0 (0.00)	2 (0.51)	1 (0.29)	0 (0.00)	0 (0.00)	11 (0.13)
**Sleep quality**								
Very good	670 (39.39)	297 (32.32)	296 (35.53)	144 (36.73)	151 (44.15)	108 (37.37)	99 (38.67)	3378 (40.16)
Good	783 (46.03)	455 (49.51)	401 (48.14)	177 (45.15)	144 (42.11)	129 (44.64)	119 (46.48)	3754 (44.63)
Average	242 (14.23)	163 (17.74)	130 (15.61)	68 (17.35)	46 (13.45)	50 (17.3)	38 (14.84)	1212 (14.41)
Bad	5 (0.29)	3 (0.33)	5 (0.60)	3 (0.77)	1 (0.29)	2 (0.69)	0 (0.00)	35 (0.42)
**Sleep time**								
≤9h	443 (26.04)	233 (25.35)	210 (25.21)	80 (20.41)	99 (28.95)	77 (26.64)	60 (23.44)	2122 (25.23)
>9&≤10h	1158 (68.08)	643 (69.97)	578 (69.39)	285 (72.70)	222 (64.91)	191 (66.09)	174 (67.97)	5695 (67.70)
>10&≤11h	94 (5.53)	41 (4.46)	43 (5.16)	25 (6.38)	19 (5.56)	19 (6.57)	20 (7.81)	526 (6.25)
>11h	1 (0.06)	1 (0.11)	1 (0.12)	1 (0.26)	0 (0.00)	0 (0.00)	0 (0.00)	17 (0.20)
**House renovation during the past year**								
Yes	369 (21.69)	207 (22.52)	207 (24.85)	91 (23.21)	79 (23.10)	59 (20.42)	45 (17.58)	1755 (20.86)
No	1326 (77.95)	709 (77.15)	624 (74.91)	299 (76.28)	262 (76.61)	230 (79.58)	210 (82.03)	6601 (78.47)
**Pet ownership**								
Yes	338 (19.87)	196 (21.33)	167 (20.05)	69 (17.60)	79 (23.10)	59 (20.42)	58 (22.66)	1502 (17.86)
No	1358 (79.84)	718 (78.13)	660 (79.23)	320 (81.63)	262 (76.61)	229 (79.24)	197 (76.95)	6851 (81.44)

**Ventilation**								
Everyday	1539 (90.48)	838 (91.19)	756 (90.76)	357 (91.07)	311 (90.94)	256 (88.58)	233 (91.02)	7556 (89.82)
1 to 3 times/week	137 (8.05)	72 (7.83)	70 (8.40)	29 (7.40)	26 (7.60)	29 (10.03)	19 (7.42)	691 (8.21)
1 to 5 times/month	14 (0.82)	4 (0.44)	4 (0.48)	3 (0.77)	3 (0.88)	4 (1.38)	3 (1.17)	87 (1.03)
Seldom	7 (0.41)	4 (0.44)	1 (0.12)	1 (0.26)	1 (0.29)	0 (0.00)	0 (0.00)	34 (0.40)
Never	0 (0.00)	0 (0.00)	0 (0.00)	0 (0.00)	0 (0.00)	0 (0.00)	0 (0.00)	3 (0.04)
**Cooking and heating fuel**								
Coal gas	147 (8.64)	79 (8.60)	66 (7.92)	34 (8.67)	23 (6.73)	21 (7.27)	19 (7.42)	692 (8.23)
Natural gas	1378 (81.01)	752 (81.83)	689 (82.71)	316 (80.61)	281 (82.16)	237 (82.01)	212 (82.81)	6738 (80.10)
Liquefied petroleum gas	78 (4.59)	42 (4.57)	38 (4.56)	21 (5.36)	19 (5.56)	14 (4.84)	15 (5.86)	458 (5.44)
Electricity	42 (2.47)	24 (2.61)	23 (2.76)	14 (3.57)	11 (3.22)	9 (3.11)	4 (1.56)	275 (3.27)
Coal	0 (0.00)	0 (0.00)	0 (0.00)	1 (0.26)	0 (0.00)	1 (0.35)	0 (0.00)	12 (0.14)
Others	4 (0.24)	1 (0.11)	0 (0.00)	0 (0.00)	0 (0.00)	0 (0.00)	0 (0.00)	8 (0.10)

†: Data are presented as mean (SD) or number (%).

‡: Missing (n): Passive smoking exposure (58); Exercise (55); Screen use (131); Sleep quality (33); Sleep time (52); House renovation during the past year (56); Pet ownership (59); Ventilation (41); Cooking and heating fuel (229).
